# Microscale Photopatterning of Through‐Thickness Modulus in a Monolithic and Functionally Graded 3D‐Printed Part

**DOI:** 10.1002/smsc.202000017

**Published:** 2021-02-11

**Authors:** Asais Camila Uzcategui, Callie I. Higgins, John E. Hergert, Andrew E. Tomaschke, Victor Crespo-Cuevas, Virginia L. Ferguson, Stephanie J. Bryant, Robert R. McLeod, Jason P. Killgore

**Affiliations:** ^1^ Materials Science and Engineering University of Colorado, Boulder Boulder CO 80309 USA; ^2^ Applied Chemicals and Materials Division (647) National Institute of Standards and Technology (NIST) Boulder CO 80305 USA; ^3^ Department of Mechanical Engineering University of Colorado, Boulder Boulder CO 80309 USA; ^4^ Department of Chemical and Biological Engineering University of Colorado, Boulder Boulder CO 80309 USA; ^5^ Department of Electrical, Computer and Energy Engineering University of Colorado, Boulder Boulder CO 80309 USA

**Keywords:** atomic force microscopy, digital light processing, functionally graded materials, mechanical gradients, 3D printing

## Abstract

3D printing is transforming traditional processing methods for applications ranging from tissue engineering to optics. To fulfill its maximum potential, 3D printing requires a robust technique for producing structures with precise 3D (*x*, *y*, and *z*) control of mechanical properties. Previous efforts to realize such spatial control of modulus within 3D‐printed parts have largely focused on low‐resolution (from mm to cm scale) multimaterial processes and grayscale approaches that spatially vary the modulus in the *x–y* plane and energy dose‐based (*E *= *I*_0_ *t*_exp_) models that do not account for the resin's sublinear response to irradiation intensity. Here, a novel approach for through‐thickness (*z*) voxelated control of mechanical properties within a single‐material, monolithic part is demonstrated. Control over the local modulus is enabled by a predictive model that incorporates the material's nonreciprocal dose response. The model is validated by application of atomic force microscopy to map the through‐thickness modulus on multilayered 3D parts. Overall, both smooth gradations (30 MPa change over ≈75 μm) and sharp step changes (30 MPa change over ≈5 μm) in the modulus are realized in poly(ethylene glycol) diacrylate‐based 3D constructs, paving the way for advancements in tissue engineering, stimuli–responsive 4D printing, and graded metamaterials.

## Graphical abstract

A gradient 3D printing platform is developed and used to fabricate monolithic parts with microscale patterning of elastic modulus. Modeling the interplay between exposure conditions and cure depth predicts a broad range of through‐thickness modulus values in digital light processing (DLP) of 3D‐printed parts. This model informs the process parameters to manufacture deliberate functionally graded materials through the part thickness.

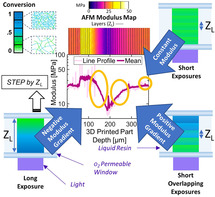

## 1 Introduction

3D printing creates complex, highly customized architectures with applications in tissue engineering, soft robotics, optics, and metamaterials.^[^ ^1^, ^2^, ^3^, ^4^ ^]^ Today, the elastic moduli of materials used for 3D printing vary from 10 s of kPa in polymers to 100 s of GPa in metals.^[^ ^5^ ^]^ However, the material properties resulting from most 3D printing methods are limited to a single property value or multiple discrete property values with limited control of spatial gradients. One of the pitfalls in the adoption of 3D printing for operational part fabrication is the lack of mechanical performance and the susceptibility of the structures to fail as compared with traditionally manufactured analogues.^[^ ^6^, ^7^ ^]^ To address these shortcomings, improvements in mechanical robustness and flaw tolerance can be obtained with functionally graded materials (FGMs).^[^ ^8^ ^]^

FGMs in the context of additive manufacturing and prototyping draw interest for their ability to mimic the changing properties found in natural structures.^[^ ^9^ ^]^ Not only do FGMs have enhanced mechanical behavior, but they are able to match the graded properties found in biological tissues; examples include the dentin–enamel junction of the tooth, the osteochondral (bone–cartilage) unit, the bone–cartilage–bone junctions of the growth plate in long bones, and the bone‐to‐tendon region, among others.^[^ ^9^, ^10^, ^11^, ^12^ ^]^ To withstand a broad range of physiological forces and prevent premature failure of structures, a method of fabricating FGMs with 3D, microscale control must be developed.^[^ ^13^ ^]^

Most efforts to fabricate FGMs using 3D printing are limited to inkjet methods that use multiple printheads to deposit different materials on the build stage.^[^ ^14^, ^15^, ^16^ ^]^ However, these methods rely on the jetting of material, which suffers from low resolution (>200 μm), slow print speed (50–150 mm h^−1^), and strict viscosity requirements.^[^ ^17^ ^]^ More recently, photopatterning and digital light processing (DLP) vat polymerization 3D printing have been used to create FGMs.^[^ ^13^, ^18^ ^]^ DLP uses a spatial light modulator (SLM) to project a series of 2D images into a resin vat to create a 3D object from a computer‐aided design file. As DLP uses a single vat of precursor solution, or resin, it is generally regarded as unsuitable for fabricating parts that vary in mechanical spatial functionality or material properties in a precise manner. Although methods that switch between multiple vats of resin were developed, these suffer from slow print speed and a limited ability to control the gradient at the material interface. DLP methods that use grayscale light intensity to 3D print FGMs were developed to have a broad range of material properties but have been limited to 2D *x–y* control in the mm–cm range.^[^ ^13^, ^19^ ^]^

We build upon recent advancements in the area of front photopolymerization kinetics to present a novel approach for predicting, verifying, and controlling conversion through the multilayer depth of a 3D‐printed part.^[^ ^20^ ^]^ We combine pioneering works focused on nonuniform photocuring in depth with our recent findings that energy dose *E* as a product of intensity *I*_0_ and exposure time *t*_exp_ alone does not adequately describe the polymerization kinetics of the radically initiated photopolymers used for 3D printing. Rather, to predict a more representative effective exposure E*, the value of *I*_0_ must be rescaled by some exponent to broadly describe kinetics for a range of light intensities such as those experienced due to light absorption through the layer or part thickness.^[^ ^7^, ^21^, ^22^ ^]^ Notably, the dependence of kinetics on effective exposure rather than energy dose expands the range of functional gradients that can be printed from a single precursor resin. Herein, we describe the experimental parameter space for 3D microscale control of mechanical properties within a monolithic part and demonstrate that gradients can be controlled through a combination of layer thickness, light intensity, and exposure time. By modeling the phenomena responsible for the intrinsic conversion variation with depth, we can impart mechanical step functions and property gradients within 3D‐printed parts in an unprecedented fashion. This variation can be utilized to achieve complex functionally graded *z*‐profiles that are unrestricted to the direction of printing. Sharp step functions up to 30 MPa over distances of ≈5 μm or gradual change in the elastic modulus of 30 MPa over 100 μm are achieved without the need for multiple materials. This micron‐scale control of the modulus is experimentally verified via nanoscale mechanical testing along the *z*‐axis of a 3D‐printed part.

**Figure 1:**
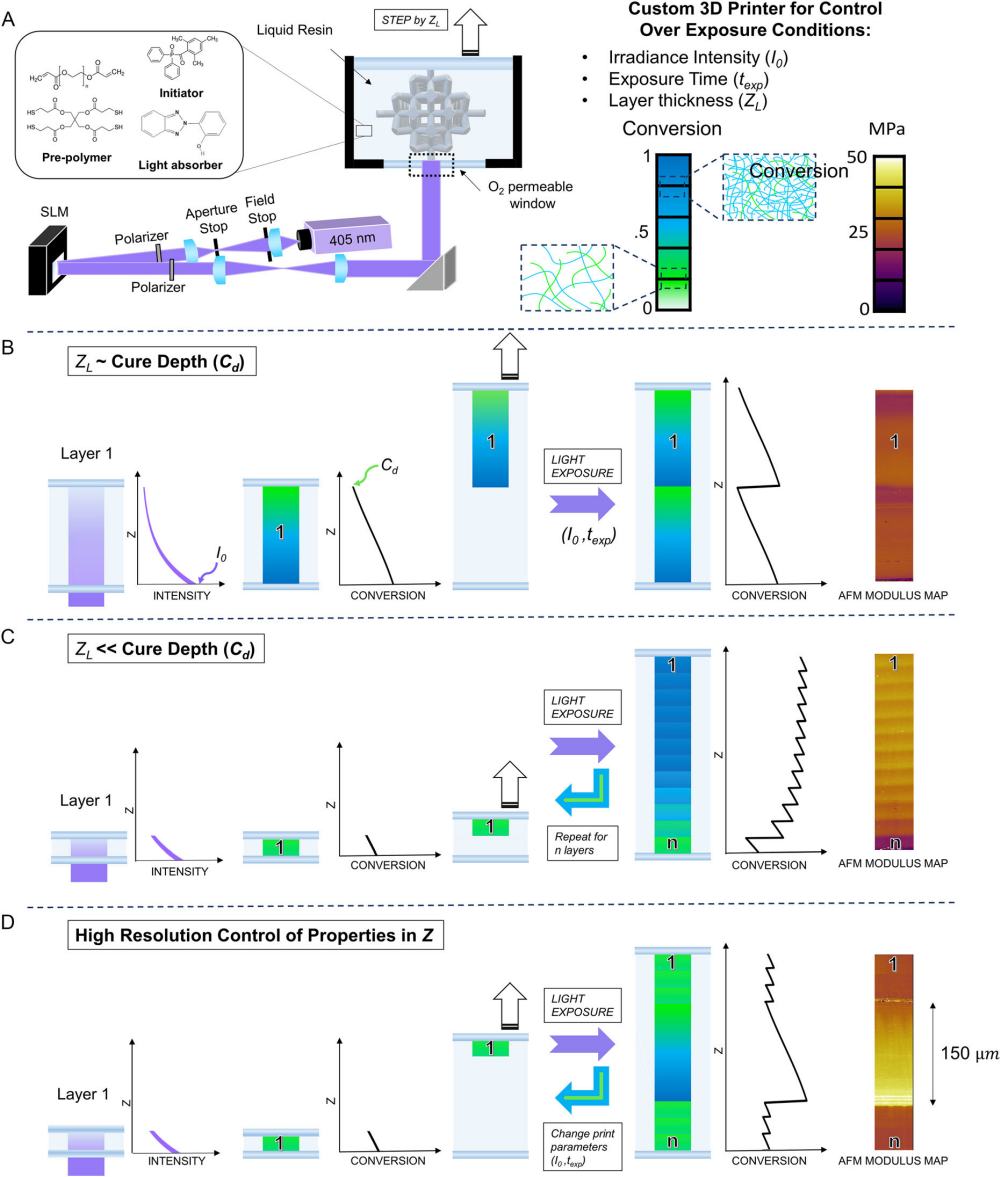
A) Custom‐built DLP printer for control over irradiance intensity (*I*_0_), exposure time (*t*_exp_), and layer thickness (*Z*_L_). These parameters are varied to produce controlled *z*‐direction conversion, which is measured by the atomic force microscopy (AFM) modulus mapping of cross‐sectioned parts. Scale bar in A applies to modulus maps of B, C, and D. B) Light absorber causes an exponential decay in light intensity along the depth of each exposed layer, leading to a decay in conversion and only a finite thickness of resin undergoes gelation. This thickness is known as the cure depth (*C*_d_). When the layer thickness (*Z*_L_) is comparable with the cure depth, there is a large change in conversion and thus modulus along the depth of each layer. C) When the layer thickness is much smaller than the cure depth, each layer experiences multiple light exposures due to light penetration in the layer‐by‐layer printing process. D) Micronscale control of properties in *z* is achieved in a single material by implementing a computational model that incorporates layer thickness, cure depth, irradiance intensity, and exposure time.

In this work, we use a model acrylate photopolymer resin that is comparable with commercial 3D printing materials to demonstrate modulus control in *z*. Our approach exploits the fact that bottom‐up DLP printing (**Figure** **1**A) has two controls on *z*‐resolution: the light penetration depth and the step size for each layer. Conversion in each printed layer varies from a maximum, just past the oxygen‐inhibited region near the print window, to a value defined by the gelation threshold for the case when layer thickness equals cure depth (Figure 1A). The cure depth is defined by the gelation threshold that is described by an exponential decay in light intensity and is governed by Beer–Lambert absorption, exposure conditions (i.e. layer thickness, light intensity, exposure time), and polymerization kinetics. When the layer thickness *Z*_L_ is approximately equal to the cure depth *C*_d_, a gradient in conversion occurs and is the same in all layers within the part (Figure 1B). When the layer thickness *Z*_L_ is less than the cure depth *C*_d_, each layer experiences overlapping light exposures from subsequently printed layers, causing an increase in conversion in the previously printed layers (Figure 1C). Modeling this process allows us to toggle between each scenario to fabricate functionally graded parts with microscale control (Figure 1D).

## 2 Modeling Effective Exposure

**Figure 2:**
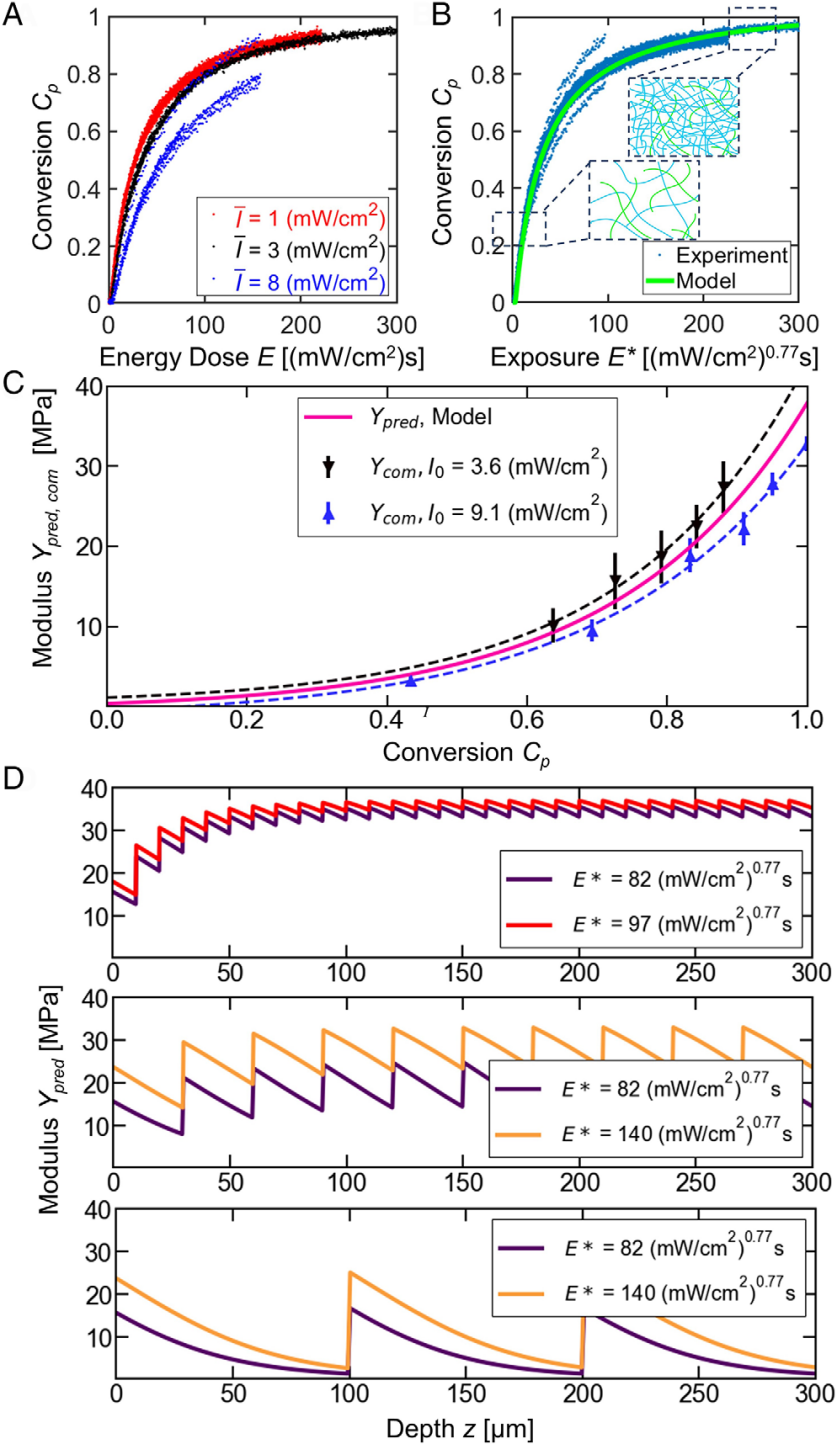
A) Acrylate monomer conversion as a function of intensity and exposure time. Reciprocity would cause these curves to overlap when plotted versus energy dose, the product of light intensity and exposure time; instead, we find that the material has a sublinear response to intensity and conversion is dependent on a scaled intensity. B) Monomer conversion as a function of the exposure condition can be expressed by a single predictive master curve (green), by accounting for the sublinear response in optical intensity. Shown is the double‐bond conversion as measured by real‐time Fourier transform infrared spectroscopy (RT‐FTIR), for three exposure intensities. The *x*‐axis is a scaled exposure dose *I*_0_^*m*^ *t,* where the scaling factor *m* = 0.77. C) Compressive Young's modulus (*Y*_com_) of bulk, optically thin layers are plotted against conversion for multiple exposure conditions, yielding a predictive curve (magenta). Error bars indicate one standard deviation. D) Through‐thickness Young's modulus variation predicted via the scaled exposure model (*Y*_pred_) for a constant energy dose (*E *= 180 mJ cm^−2^) while varying layer thickness *Z*_L_ between 10, 30, and 100 μm and different effective exposures. The different intensity and time combinations give differing exposures (E*), which control the positive gradient achieved by the 10 μm layers (red vs purple) and the negative gradient achieved by a 100 μm layer (yellow vs purple). In both cases, a higher effective exposure causes a steeper gradient in the positive direction (red, *Z*_L_ = 10 μm) and negative direction (yellow, *Z*_L_ = 100 μm). The exposure conditions chosen are the same as the ones used for experiments in Figure 3.

To model the modulus profile in a 3D‐printed part from a series of exposures and layer thicknesses, the relationship between conversion, accumulated energy dose, and cure depth must first be described. However, earlier models often simplify the photochemical reactions by assuming that resins are “reciprocal,” meaning that the resin response depends only on the product of intensity (*I*_0_) and time (*t*_exp_). This assumption often fails for radically initiated photopolymers.^[^ ^23^ ^]^ While primary photochemical reactions such as absorbance and initiator cleavage follow the first‐order scaling of reciprocity, radical polymerizations are known for their sublinear dependence on light intensity due to subsequent bimolecular radical termination.^[^ ^24^, ^25^ ^]^ Thus, kinetics in photopolymerization as a function of light intensity *I*_0_ and exposure time *t*_exp_ often cannot be described solely by optical energy dose *E *= *I*_0_ *t*_exp_, particularly at the high intensities and small volumes required for 3D printing.^[^ ^26^ ^]^ Instead, our previous work showed that polymerization kinetics in acrylate‐based resins are related to light intensity by a power law where conversion *C*_p_ is proportional to the product of intensity raised to a power *m* and time *t*_exp_, 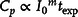, thus giving effective exposure *E* ^*^ = *I*_0_^*m*^ *t*_exp_.^[^ ^7^ ^]^ For a given position in *z*, the polymerization regime is preceded in time by an oxygen inhibition regime, which determines the intensity and exposure duration required to overcome the inhibition threshold. In traditional models of DLP and stereolithography (SLA), the energy required to overcome inhibition and reach gelation is known as the critical energy dose, *E*_c_, where *E*_c_ = *I*_0_ *t*_c,_ and *t*_c_ is the critical time for gelation. Our previous work showed that rather than using the traditional *E*_c_, the exposure conditions to overcome the gelation threshold can more accurately be modeled using a scaled critical exposure *E*_c_^*^ = *I*_0_^*n*^ *t*_c_; this leads to a more representative working curve that describes cure depth *C*_d_ as a function of *E*_c_^*^, absorbance, irradiance intensity, and exposure time.^[^ ^7^ ^]^ The importance of differentiating between the *n* in *E*_c_^*^ and *m* in *E* ^*^ is further described in the study by Uzcategui et al.^[^ ^7^ ^]^ To determine the value of *n*, curing kinetics were studied using RT‐FTIR to correlate *C*_p_ with *I*_0_ and *t*_exp_ as established previously.^[^ ^7^ ^]^ Earlier works have simplified RT‐FTIR measurements by carrying them out on resins without added photoabsorbers. However, the photoabsorber used in this study significantly affected the polymerization kinetics of the resin and thus was incorporated into the RT‐FTIR resin formulation to better represent the kinetics in the 3D‐printed parts. To account for the presence of the absorber, we used the mean of the Beer–Lambert exponential decay function to get mean intensity through depth. Further details of these effects can be found in Supporting Information. The scaling factor *n* = 0.53 was found by measuring *E*_c_ for multiple exposure conditions, ultimately resulting in *E*_c_^*^ = 0.96 [(mW cm^−2^)^0.53^s]. Conversion is plotted as a function of time and intensity using a scaling factor *m* = 1 (i.e., assuming reciprocity) and using a common energy dose (*E *= *I*_0_^*1*^ *t*_exp_) in **Figure** **2**A. Conversely, the same data can be collapsed on to a single master curve by adjustment of *m.* To find *m*, the linear portion of the conversion curve was extracted for each exposure condition. The value of *m* was varied to minimize the error between the initial slopes, resulting in a best fit master curve with *m *= 0.77. The scaled data were fit to an empirical relationship with each regime scaled to appropriate light intensities (Figure 2B). A third‐order polynomial function most accurately captured the shape of the master curve, which was not achieved from steady‐state approximation. The master curve for conversion was fit to

(1) $$C_{p}=\frac{x^{3}+ax+b}{x^{3}+cx+d}$$

where

(2) $$x=I_{0}^{m}(t_{\exp}-\frac{E_{c}^{*}}{I_{0}^{n}})$$

and *a* = 2.32 × 10^5^, *b* = −1.38 × 10^5^, *c *= 2.20 × 10^5^, and *d* = 6.35 × 10^6^. Previous work demonstrated that modulus scales exponentially with conversion.^[^ ^27^ ^]^ Thus, a second master curve was fit to Young's modulus (*Y*_com_) as a function of *C*_p_ to derive a predictive relationship for *Y*_pred_ *(C*_p_ *)* defined by

(3) $$Y_{pred}(C_{p})=Y_{c}\exp(\beta C_{p})+Y_{d}$$

where *Y*_c_ = 0.88, *β* = 3.77, and *Y*_d_ = 0.52 are fitting parameters. To determine the relationship between conversion and modulus, a series of optically thin and thus nominally homogeneous layers (*Z*_L_ = 2 mm, no photoabsorber added) were printed with varying exposure conditions and evaluated for Young's modulus under compression. The results (Figure 2C) indicate that local modulus can be approximated in our model solely as a function of local conversion. This model also has the advantage of being simple to invert mathematically, unlike one based on intensity. The scaled model was inverted and applied to a diverse set of printing conditions to investigate the impact of layer thickness *Z*_L_, energy dose *E*, and effective exposure E* on the through‐thickness modulus of the part. Figure 2D shows the impact of using *E*, while varying *Z*_L_ and E*. Notably, the scaled model highlights that using the same energy dose but different intensity and time, different E*, has a direct impact on the modulus profile of each layer and the modulus profile across multiple layers. This reveals that there is an additional control parameter that can be exploited in nonreciprocal materials to create functional gradients not possible with reciprocal materials where the cross‐sectional modulus profile would depend only on *E*. Keeping energy dose constant, lower intensity leads to sharper negative mechanical gradients in layer thicknesses that are close to the cure depth *C*_d_ and leads to sharper positive gradients across layer thicknesses that are considerably smaller than the cure depth *C*_d_ (Figure 2D). Importantly, this polymerization kinetic behavior is common in most acrylate resins, so our method is applicable to a diverse set of thermosets where local stiffness scales with local conversion.

## 3 Experimental Verification of Effective‐Exposure Model

**Figure 3:**
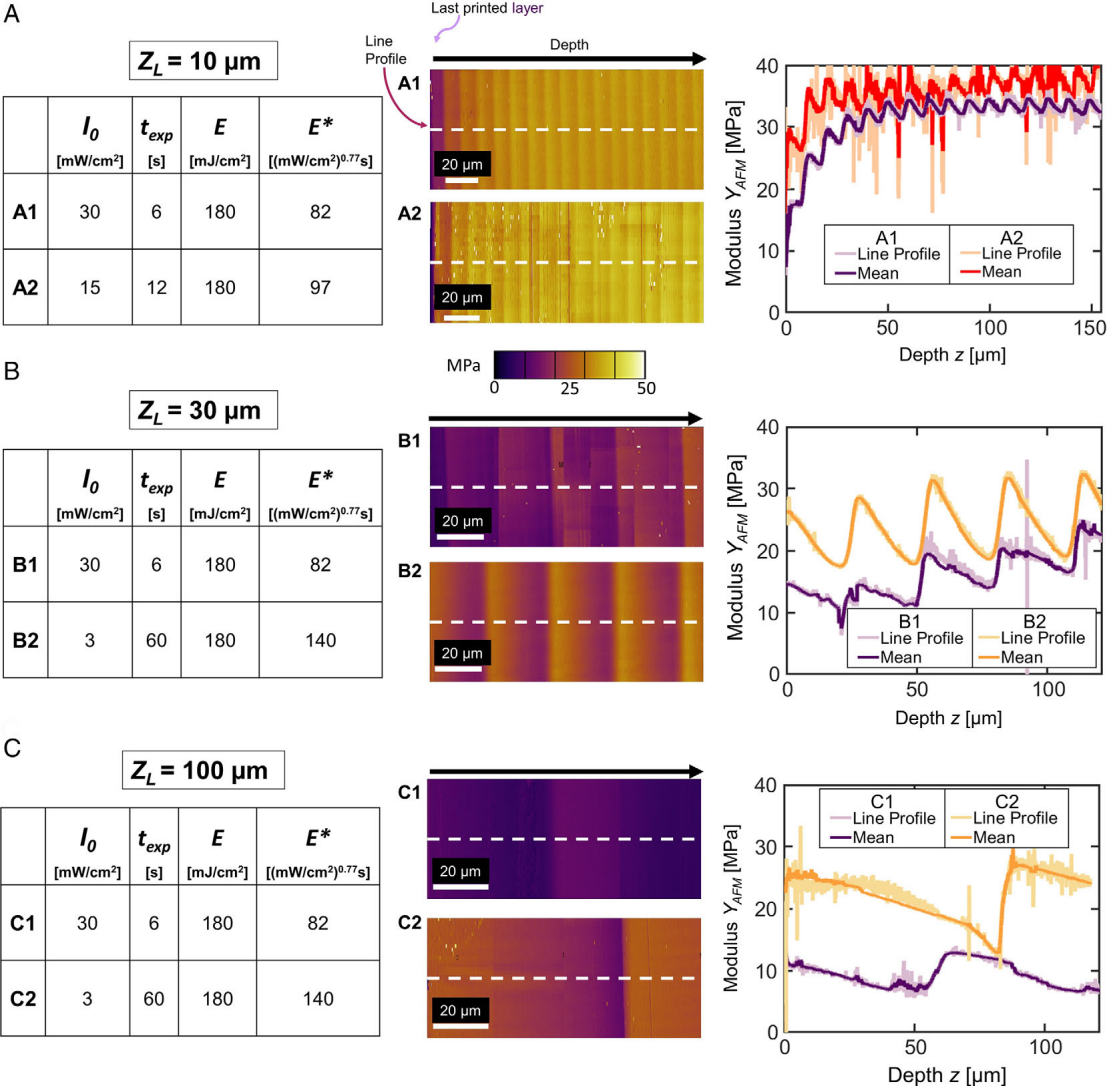
AFM maps of through‐thickness Young's modulus of 3D‐printed structures, demonstrating intrinsic mechanical heterogeneity that arises from the resin's sublinear response to intensity, light absorption, repeated exposure, and species diffusion. The white dashed line indicates where the line profile was taken. The energy dose was kept constant for all samples *(E* = 180 mJ cm^−2^ *).* A) Map of *Y*_AFM_ for a structure 3D printed with *Z*_L_ = 10 μm, where the intensity and time were varied to probe the effect of sublinear intensity scaling. Lower intensity and longer time (red) lead to both a higher mean modulus and a steeper positive through‐thickness gradient. B) Map of *Y*_AFM_ for a structure printed with *Z*_L_ =30 μm, showing a similar steepening of the positive gradient (orange) to A. C) Map of *Y*_AFM_ for *Z*_L_ = 100 μm, where the gradient is reversed and becomes steeper in the negative direction as the intensity is lowered and the time is increased. The graphs depict the line profiles (light color) and mean (dark color) of the AFM elastic modulus through thickness. We attribute spikes in the data to contaminants and sample preparation artifacts.

To test the efficacy of our scaled effective exposure model, structures were fabricated using the custom DLP system shown in Figure 1A, applying an equivalent energy dose *E*, while varying layer thickness *Z*_L_ between 10, 30, and 100 μm and varying effective exposure E* between 82 (mW cm^−2^)^0.77^s, 97 (mW cm^−2^)^0.77^s, and 140 (mW cm^−2^)^0.77^s. Equivalent‐dose structures with *Z*_L_ *=* 10 μm and E* = 140 (mW cm^−2^)^0.77^s consistently over‐adhered to the window during printing, so E* = 97 (mW cm^−2^)^0.77^s was used as the dose‐equivalent condition for comparison at this smaller layer thickness. The through‐thickness modulus variation in *z* was experimentally determined using AFM nanomechanical measurements on ultra‐cryomicrotomed cross sections of DLP 3D‐printed parts. **Figure** **3** shows the through‐thickness AFM modulus (*Y*_AFM_) variation in equivalent energy dose structures that were printed with different exposure times, exposure intensities, and layer thicknesses. The combined theoretical and experimental findings reveal that a higher *E* ^*^ leads to a steeper mechanical gradient at ≈100 μm length scales. A steeper positive gradient is achieved with higher *E* ^*^ and *Z*_L_ << *C*_d_ and a steeper negative gradient is achieved with higher *E* ^*^ and *Z*_L_ ≈*C*_d_. A comparison of Figure 2 and 3 highlights that effective exposure reliably predicts final part modulus trends. For *Z*_L_ = 10 μm and exposures E* = (82, 97) (mW cm^−2^)^0.77^s, the Young's modulus averaged through the part thickness was (31.4 ± 5.2, 34.4 ± 4.2) MPa and the average experimental AFM modulus was (31.4 ± 4.1, 34.5 ± 6.3) MPa. For *Z*_L_ = 30 μm and exposures E* = (82, 140) (mW cm^−2^)^0.77^s, the average predicted modulus was = (18.2 ± 3.8, 26.8± 4.1) MPa and the average experimental AFM modulus was = (19.4 ± 4.7, 23.8 ± 4.3) MPa. For *Z*_L_ = 100 μm and exposures E* = (82, 140) (mW cm^−2^)^0.77^s the average predicted modulus was = (6.2 ± 4.4, 11.0 ± 6.6) MPa and the average experimental AFM modulus was = (9.5 ± 2.2, 22.2 ± 3.7) MPa. A quantitative comparison between the accuracy of scaled and unscaled exposure models is included in Supporting Information (Figure S1 and S2, Supporting Information). Our findings show that increasing effective exposure leads to increased Young's modulus throughout the part and increasing layer thickness leads to decreased modulus throughout the part (Figure 3). The latter is consistent with findings from Zhao et al. where the mean modulus of parts printed with *Z*_L_ = 100 μm was higher than of those printed with *Z*_L_ = 150 μm.^[^ ^21^ ^]^ We note that this dependence on layer thickness is dominated by overlapping light exposures as *Z*_L_ decreases, which is noted in the study by Zhao et al. and is discussed above. This effect is less pronounced in the case where *Z*_L_ = 100 μm because the cure depth of the material for this *I*_0_ and *t*_exp_ is *C*_d_ ≈125 μm, causing minimal overlapping exposure between layers (Figure 3D).

To ensure the accuracy of experimental layer thickness and modulus values for *Z*_L_ = 100 μm, we used nanoindentation (NI) as a complementary nanomechanical characterization tool (Figure S3, Supporting Information). The NI and AFM results are consistent: *Y*_NI_ ranged from 9.6 to 13.3 MPa and *Y*_AFM_ ranged from 7.5 to 13.1 MPa. In both NI and AFM results, *Z*_L_ = 100 μm, E* = 82 (mW cm^−2^)^0.77^s case shows a ≈30 % shrinkage during postprocessing because of the removal of unreacted monomer in the conversion gradient of the layer.

Of note, the discrepancy between *Y*_pred_ and *Y*_AFM_ increased at a higher layer thickness. This is explained by the effect that high exposure time and low conversion has on localized diffusion of monomer and oligomers in the partially gelled network. Canal and Peppas, and Muralidharan et al. demonstrated that mesh size can be prescribed by exposure conditions and thus conversion.^[^ ^28^, ^29^ ^]^ Muralidharan showed that 100% conversion yielded a mesh size of ≈1.3 nm, whereas ≈13.5% conversion yielded a mesh size of ≈11 nm in the same acrylate‐based resin used herein. Transport of monomer occurs when the hydrodynamic radius is smaller than the mesh size of the network.^[^ ^29^ ^]^ Estimating the monomer diffusivity *D* based on the diffusivity of a 1000 g mol^−1^ PEGDA molecule (*D *= 72 μm^2^ s^−1^), the characteristic diffusion length ($\sqrt{Dt}$) is ≈19 μm for the 5 s wait time between each exposure.^[^ ^29^ ^]^ Therefore, diffusion occurs on a relevant length scale during the wait time between layers, a phenomenon that was not accounted for in our model but would result in a higher‐than‐predicted modulus. This finding is congruent with compression testing findings in the supplement and the work of Fiedler–Higgins et al., where modulus increased by an order of magnitude following multiple diffusion‐exposure cycles.^[^ ^30^ ^]^ We also note that our experimental results indicate a gradient in *Y*_AFM_ rather than a sharp change between layers predicted by the model partially due to the inhibitory effects of dissolved oxygen; this is consistent with a recent study performed by Gojzewski et al.^[^ ^31^ ^]^ Moreover, these gradients likely serve to dissipate energy and toughen interfaces formed between layers.^[^ ^32^, ^33^ ^]^ The model is able to predict microscale *Y*(*z*) during printing over process conditions where transport is negligible, while predicting relative modulus change (e.g., step changes, gradients) in the presence of significant diffusion and accounting for effective exposure.

## 4 Programming of Functionally‐Graded Modulus Profiles

**Figure 4:**
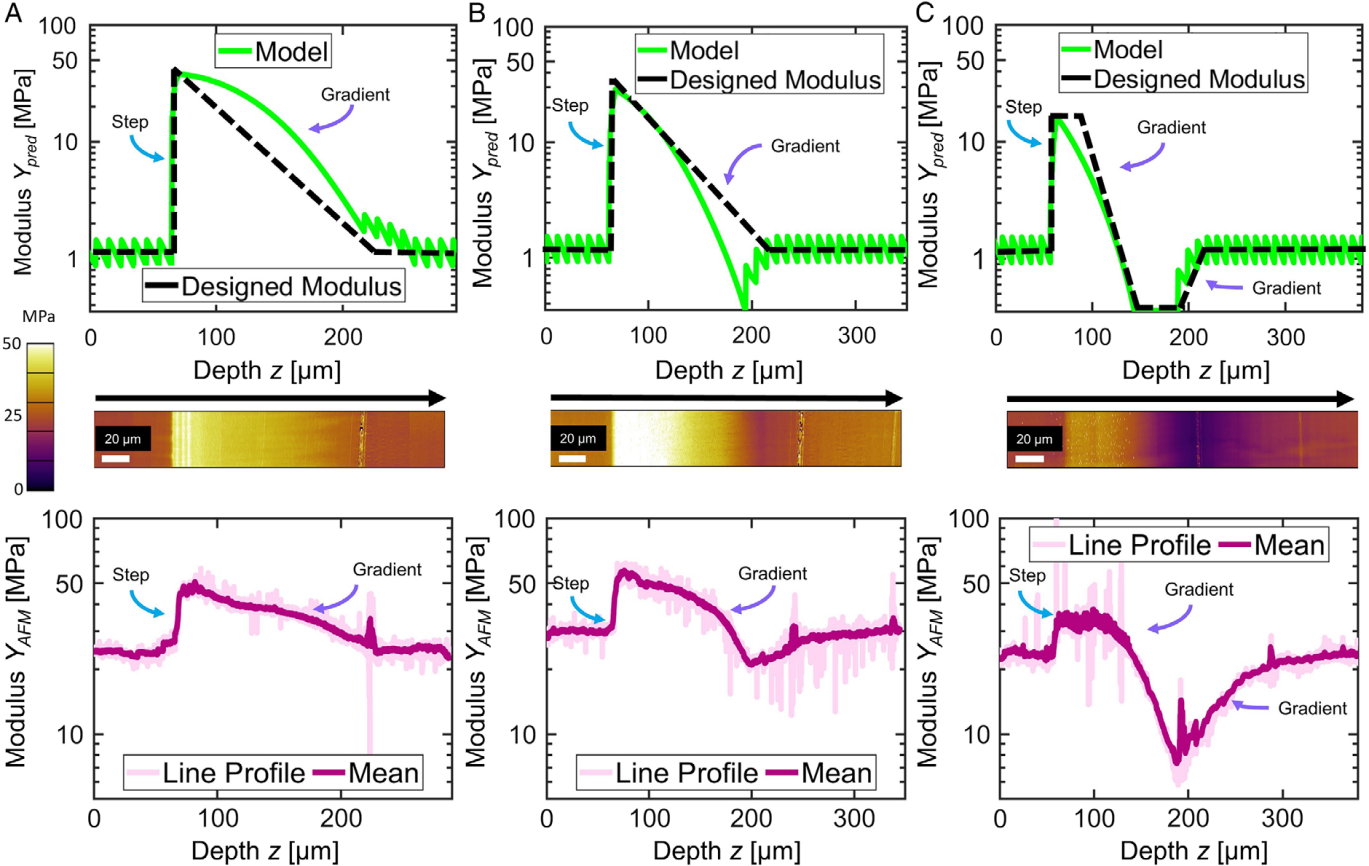
AFM modulus maps of 3D‐printed monolithic structures, showing that the optimal choice of exposure conditions, as informed by our scaled model, enables micronscale through‐thickness patterning of modulus. The light pink data is a line profile of the AFM modulus map (*Y*_AFM_), the dark pink line is the mean of the AFM map for a vertical column, and the green line is the programmed modulus (*Y*_pred_) based on the model that does not account for transport. A) Modulus as a function of depth showing three regions of programmed stiffness. A step function from 26–50 MPa, and a 150 μm continuous gradient to 26  MPa. B) Three regions of programmed stiffness with a step function from 28–60 MPa and a 100 μm continuous gradient to 28 MPa. C) Four regions of programmed stiffness with a step function from 26–35 MPa, a 75 μm continuous gradient to 6 MPa, and a 75 μm continuous gradient to 26 MPa.

With the predictive ability of the model established, next, the model was used to inform how exposure conditions (i.e., *Z*_L_ *, I*_0_ *, t*_exp_) can be calculated from a designed modulus profile *Y*(*z*) to exercise 3D control over the fabrication of monolithic structures with programmed step functions and functional gradients in modulus. By combining Equation (1) and (3), Y(*z*) can be programmed to create monolithic 3D‐printed parts with regions of low and high modulus, step functions, and gradients. With the model and custom 3D printer, which allows dynamic adjustment of layer thickness and the effective exposure on each printed layer, the sharpness of the step functions and the length scale of the gradients can readily be programmed. **Table** **1** shows the dynamic exposure conditions used for **Figure** **4**. The first example of patterned Young's modulus (Figure 4A) is a structure designed to have four distinct mechanical regions: a low modulus region, a low‐to‐high modulus step, a high‐to‐low modulus gradient, and a second low modulus region. Using the conditions in Table 1, the structure in Figure 4A achieved a *Y*_AFM_ step increase from 26 to 50 MPa over just 7 μm. The 50 MPa modulus then decreased in a gradient back to 26 MPa over a 150 μm distance, after which the lower modulus was maintained. Notably, the gradient, which is longer than cure depth *C*_d_, is achieved by introducing an interlayer sawtooth with decaying mean intensity to the intralayer sawtooth defined by *C*_d_. Likewise, the thin‐layered sawtooth configuration is also the primary means of achieving pseudoconstant modulus value over those defined regions.

**Table 1:** Exposure conditions used to fabricate parts in Figure 4

Sample	*I*_o_ [mW cm^−2^] All layers	Z_L1_ [μm] 100 layers	*t*_exp1_ [s] 100 layers	Z_L2_ [μm] 1 layer	*t*_exp2_ [s] 1 layer	Z_L3_ [μm] 5 layers	*t*_exp3_ [s] 5 layers	Z_L4_ [μm] 100 layers	*t*_exp4_ [s] 100 layers
4A	30	10	0.6	146	6	1	6	10	0.6
4B	30	10	0.6	126	2.4	1	2.4	10	0.6
4C	30	10	0.6	126	1.2	1	1.2	10	0.6

Our second example (Figure 4B) seeks to shrink the length scale of the gradient region to 100 μm, which is smaller than *C*_d_ for the conditions in Figure 4A. The gradient control is achieved by exploiting the relationship between *I*_0,_ *t*_exp_, and *C*_d_. To achieve the sharper gradient requires a shorter exposure time and a calculated undershooting of the target modulus. By applying the conditions in Table 1 for Sample 4B, this sharper gradient is experimentally realized to decrease from *Y*_AFM_ = 60 MPa to *Y*_AFM_ = 28 MPa over 105 μm. Although the exposure time in Figure 4B is lower than exposure time in Figure 4A, we achieve a larger modulus step due to the greater influence of monomer diffusion for *Z*_L2_ = 1 μm, *t*_exp2_ = 2.4 s exposure, where conversion is low as compared with in *Z*_L2_ = 1 μm, *t*_exp2_ = 6 s exposure, where conversion is high.

Our final example of the patterned modulus is a structure with three distinct constant modulus regions and both positive and negative 75 μm gradients (Figure 4C). The exposure pattern exhibits an intermediate modulus region, a step‐to‐high modulus region, a gradient‐to‐low modulus region, and a gradient back‐to‐intermediate modulus region. This process shown in Table 1 achieved the bidirectional gradients and multiple modulus levels by further reducing exposure time to *t*_exp2_ = 1.2 s for the second and third layers compared with processing for Figure 4B. Overall, the model shows a powerful ability to produce deliberate *z*‐direction modulus control through straightforward process parameter variation. This method is uniquely appealing for applications that require micronscale mechanical patterning, which is relevant to tissue engineering, graded metamaterials, and 4D printing.^[^ ^13^, ^34^, ^35^, ^36^ ^]^

## 5 Conclusion

In summary, we describe a concept and process that greatly extends the capability of DLP printing for high‐resolution digital manufacturing of parts with complex shapes and programmable functional gradients. We report micronscale, multidimensional control of modulus in 3D‐printed parts through a model‐informed, experimentally validated approach. Both the process simplicity and low detriment on printing speed stand in contrast with current methods for achieving high‐resolution 3D mechanical control which relies on multiple precursors and an ability to exchange them. We further illustrate the unique advantage of applying a robust computational model that uses scaled exposure (E* = *I*_0_^*m*^ *t*_exp_) as opposed to energy dose (*E *= *I*_0_^*1*^ *t*_exp_) to pattern the modulus in the *z*‐direction. The scaled exposure model reveals direct control over inter‐ and intralayer gradients, which provides positive and negative mechanical gradient control, respectively. Our findings complement prior works using energy dose‐dependent photopolymerization kinetics and demonstrating *x*–*y* patterning, thus forming the final piece of the puzzle for 3D property control. We note that the model also illuminates the effect of transport in areas of low conversion, suggesting further studies into the effect of diffusion on final part modulus. In principle, the method described herein can be applied to any material with a well‐behaved master curve (Equation (1)), providing new understanding and capabilities to functional DLP printing.

## 6 Experimental Section

6.1

6.1.1

##### Materials

Poly(ethylene glycol) diacrylate (PEGDA 700, Aldrich), pentaerythritol tetrakis(3‐mercaptopropionate) (PETMP >95%, Aldrich), diphenyl(2,4,6‐trimethylbenzoyl)phosphine oxide (TPO, 97 %, Aldrich), and 2‐(2‐hydroxyphenyl)‐benzotriazole derivative (TinuvinCarboProtect, BASF Company) were used.

##### Custom‐Built DLP System

This study used a custom‐built DLP system for 3D printing. This DLP used a 405 nm light‐emitting diode (LED) (SOLIS‐405C, Thorlabs) as a light source and SLM (1920 × 1152 Analog SLM, Meadowlark Optics) as the programmable mask. (Figure 1A) An acrylate‐functionalized glass slide was used as the build stage. Glass slides were functionalized according to the Gelest Silanation Protocol using 3‐(trimethoxysilyl)propyl methacrylate) (98% Aldrich) as the silane. A neutral density filter (optical density OD = 4) was used with index‐matched immersion oil (Type B, Cargille Laboratories Inc.) to prevent back scattering. The polydimethylsiloxane print window was prepared using the Sylgard 184 Silicone Elastomer Kit (DOW CORNING) mixed at a 10:1 resin:hardener ratio. The mixture was degassed and cured between two glass slides with a 1 mm spacer at room temperature for 24 h.

##### Conversion Measurement

PEGDA 700 and PETMP were mixed at 99:1 ratio by weight with 0.85 wt% TPO as a photoinitiator to enable free radical photopolymerization of the acrylate groups under UV–vis exposure and 0.8 wt% TinuvinCarboProtect photoabsorber. RT‐FTIR was utilized for kinetic analysis using a Nicolet 6700 FTIR spectrometer (Madison, WI) with a KBr beam splitter and an mercury–cadmium–telluride A (MCT/A) detector. The resin sample was placed between glass slides with 127 μm spacers. A 405 nm LED was used (M405L2‐C5, Thorlabs) to irradiate the sample. The light intensities used for this study were 3.5, 11.6, and 30 mW cm^−2^. To account for the presence of light absorbers, the light intensities were averaged through a depth of 127 μm using the Beer–Lambert law. Conversion was monitored by the disappearance of the double bond (C═C) peak of acrylate at 6200 cm^−1^ in the near‐IR range. This experiment was repeated with a resin that did not contain photoabsorbers for kinetic comparison.

##### Bulk Mechanical Testing

Unconfined compression testing was conducted (MTS; Eden Prairie, MN; 250 N load cell) on dry cylinder‐like structures fabricated using our custom DLP 3D printer. Structures (*n* = 5 per group) were measured for final dimensions (diameter: 2.358 ± 0.01 mm; height: 1.9 ± 0.035 mm) and subjected to a 3 mN preload followed by a constant displacement rate of 0.03 mm s^−1^ until failure. Deviations from the desired printed dimensions and cylindrical shape were taken into account by calculating stress and strain using measured dimensions and calculating the mean cross‐sectional area for each sample. The stress and strain data were analyzed, assuming a Poisson's ratio of 0.5, to yield the true, or “Young's”, modulus, which is the slope of the linear region of points of the true stress versus true strain curve.^[^ ^37^ ^]^

##### 3D Prints for Model Validation and Z‐control Demonstration

Trapezoidal structures were fabricated at different exposure conditions to validate a model for the effect of light intensity, exposure time, and layer thickness on through‐thickness elastic modulus. The trapezoid shape was chosen to aid in the sample preparation process. An image of a trapezoid was exposed using a 405 nm LED at *I*_0_ = 30 mW cm^−2^ for *t*_exp_ = 6 s at layer thicknesses of 10, 30, and 100 μm, at *I*_0_ = 3 mW cm^−2^ for *t*_exp_ = 60 s at layer thicknesses of 30 and 100 μm, and at *I*_0_ = 15 mW cm^−2^ for *t*_exp_ = 12 s at a layer thickness of 10 μm to create structures with a final thickness of 2 mm. Structures with a programmed through‐thickness modulus profile were exposed with changing exposure conditions (*Z*_L_ and *t*_exp_) throughout key layers of the print. All of the structures were irradiated for *t*_exp_ = 0.6 s at *Z*_L_ = 10 μm for the first 100 layers. In the structure from Figure 4A, the stage was subsequently moved by 146 μm followed by five 1 μm exposures at *t*_exp_ = 6 s. In the structure from Figure 4B, the stage was moved by 126 μm followed by five 1 μm exposures at *t*_exp_ = 2.4 s. In the structure, from figure 4C, the stage was moved by 126 μm followed by five 1 μm exposures at *t*_exp_ = 1.2 s. In all cases, the remaining layers were irradiated for *t*_exp_ = 0.6 s.

##### AFM Sample Preparation and Testing

AFM (AFM, Cypher, Asylum Research) was used in the fast force mapping (FFM) mode to measure the elastic modulus along the surface of cross‐sectioned 3D‐printed parts. A cryo‐ultramicrotome (Leica, EM FC7) was used to generate a nanometer‐smooth cross section of the printed structures required for accurate FFM. Compression testing specimens of the fully polymerized material were periodically referenced to calibrate small changes in the tip radius. The indentation rate was 300 Hz, the max force set point was 60 nN, and the force distance was 800 nm. The force curves were analyzed through a fitting routine using the Johnson Kendall Roberts (JKR) model to extract the elastic modulus while considering adhesion effects. AFM scans (30 μm × 30 μm) were digitally stitched to produce a measurement of the photopattern of the printed part. For visualization of the modulus variation, cross‐sectional profiles were obtained from the center row of the stitched image and compared with the arithmetic mean taken across all pixels along the vertical, *y*, axis.

##### NI Sample Preparation and Testing

Instrumented indentation testing was conducted on a Hysitron TI‐950 Triboindenter (Bruker, Eden Prairie MN) using a 5 μm (nominal), 4.032 μm (calibrated), radius cono–spherical probe and XZ‐500 extended displacement stage. Prior to testing, the 100 μm‐thick layered sample was stored with a desiccant for 7 days. Upon removal from the container, two layers in the center of the sample were identified as the region of interest for testing. A 4 ×11 array of indents with 10 μm spacing was placed to span two central 100 μm layers. The starting point of each row was staggered by 2.5 μm to create effective spacing of 2.5 μm across the width of the layer and avoid overlapping of indentation stress fields. Indents were conducted using a load function in displacement control as follows: a surface find was conducted with a 2 μN preload, the probe was then fully retracted by 1000 nm before reapproaching the sample at 100 nm s^−1^ and testing to a peak displacement of 1250 nm relative to the initial surface find. The peak displacement was held for 5 s (i.e., due to no viscoelastic creep observed during longer hold times) before unloading at 100 nm s^−1^ to a height of 1000 nm above the initial surface find. The 1000 nm lifts pre‐ and postindentation allowed the full adhesion response to be captured for analysis. For each NI test, the region of the unloading curve between the initial unloading point and the point of maximum adhesion was fit to the nano‐JKR model, as described by Kohn and Ebenstein,^[^ ^38^ ^]^ and Poisson's ratio of 0.5 was assumed to calculate Young's modulus.

## Conflict of Interest

The authors declare no conflict of interest.

## Supporting information

Supplementary Material

## References

[1] S. V. Murphy , A. Atala , Nat. Biotechnol. 2014, 32, 773. 25093879 10.1038/nbt.2958

[2] E. A. Aisenbrey , A. Tomaschke , E. Kleinjan , A. Muralidharan , C. Pascual-Garrido , R. R. Mcleod , V. L. Ferguson , S. J. Bryant , Macromol. Biosci. 2017, 18, 1700267. 10.1002/mabi.201700267 PMC5959280 29266791

[3] G. J. Pagan-Diaz , X. Zhang , L. Grant , Y. Kim , O. Aydin , C. Cvetkovic , E. Ko , E. Solomon , J. Hollis , H. Kong , Adv. Funct. Mater. 2018, 28, 1801145.

[4] J. A. Jackson , M. C. Messner , N. A. Dudukovic , W. L. Smith , L. Bekker , B. Moran , A. M. Golobic , A. J. Pascall , E. B. Duoss , K. J. Loh , C. M. Spadaccini , Sci. Adv. 2018, 4, eaau64419. 10.1126/sciadv.aau6419 PMC6286172 30539147

[5] H. Li , C. Tan , L. Li , Mater. Des. 2018, 159, 20.

[6] L. R. Sbriglia , A. M. Baker , J. M. Thompson , R. V. Morgan , A. J. Wachtor , J. D. Bernardin , in Conf. Proc. of the Society for Experimental Mechanics Series, Springer, New York, 2016, pp. 205–214.

[7] A. C. Uzcategui , A. Muralidharan , V. L. Ferguson , S. J. Bryant , R. R. McLeod , Adv. Eng. Mater. 2018, 20, 1800876. 30766445 10.1002/adem.201800876 PMC6370025

[8] H. Gao , B. Ji , I. L. Jäger , E. Arzt , P. Fratzl , Proc. Natl. Acad. Sci. USA 2003, 100, 5597. 12732735 10.1073/pnas.0631609100 PMC156246

[9] Z. Liu , M. A. Meyers , Z. Zhang , R. O. Ritchie , Functional Gradients and Heterogeneities in Biological Materials: Design Principles, Functions, and Bioinspired Applications, Vol. 88, Elsevier, Amsterdam 2017, pp. 467–498.

[10] G. M. Genin , S. Thomopoulos , The Tendon-to-Bone Attachment: Unification Through Disarray, Vol. 16, Nature Publishing Group, London 2017, pp. 607–608. 10.1038/nmat4906 PMC5575797 28541313

[11] S. E. Campbell , V. L. Ferguson , D. C. Hurley , Acta Biomater. 2012, 8, 4389. 22877818 10.1016/j.actbio.2012.07.042

[12] J. L. Williams , P. D. Do , J. David Eick , T. L. Schmidt , J. Orthop. Res. 2001, 19, 1043. 11781003 10.1016/S0736-0266(01)00040-7

[13] X. Kuang , J. Wu , K. Chen , Z. Zhao , Z. Ding , F. Hu , D. Fang , H. J. Qi , Sci. Adv. 2019, 5, eaav5790. 31058222 10.1126/sciadv.aav5790 PMC6499595

[14] B. Derby , Annu. Rev. Mater. Res. 2010, 40, 395.

[15] J. A. Lewis , Adv. Funct. Mater. 2006, 16, 2193.

[16] D. Kokkinis , F. Bouville , A. R. Studart , Adv. Mater. 2018, 30, 1705808. 10.1002/adma.201705808 29337394

[17] J. Mueller , D. Courty , M. Spielhofer , R. Spolenak , K. Shea , 3D Print. Addit. Manuf. 2017, 4, 193.

[18] L. M. Cox , A. K. Blevins , J. A. Drisko , Y. Qi , Y. Ding , C. I. Fiedler-Higgins , R. Long , C. N. Bowman , J. P. Killgore , Adv. Eng. Mater. 2019, 21, 1900578. 10.1002/adem.201900578 PMC13273708 42317788

[19] H. Yin , Y. Ding , Y. Zhai , W. Tan , X. Yin , Nat. Commun. 2018, 9, 1. 30291242 10.1038/s41467-018-06685-1 PMC6173746

[20] A. Vitale , J. Cabral , Materials 2016, 9, 760.28773881

[21] Z. Zhao , D. Wu , H. Sen Chen , H. Jerry Qi , D. Fang , Addit. Manuf. 2020, 35, 101420.

[22] J. Wu , Z. Zhao , C. M. Hamel , X. Mu , X. Kuang , Z. Guo , H. J. Qi , J. Mech. Phys. Solids 2018, 112, 25.

[23] J. W. Wydra , N. B. Cramer , J. W. Stansbury , C. N. Bowman , Dent. Mater. 2014, 30, 605. 24674341 10.1016/j.dental.2014.02.021 PMC4077402

[24] J. H. Lee , R. K. Prud'homme , I. A. Aksay , J. Mater. Res. 2001, 16, 3536.

[25] A. Boddapati , S. B. Rahane , R. P. Slopek , V. Breedveld , C. L. Henderson , M. A. Grover , Polymer 2011, 52, 866.

[26] C. I. Fiedler-Higgins , L. M. Cox , F. W. DelRio , J. P. Killgore , Small Methods 2019, 3, 1800275. 31289746 10.1002/smtd.201800275 PMC6615886

[27] J. Wang , X. Mu , D. Li , C. Yu , X. Cheng , N. Dai , Adv. Eng. Mater. 2019, 2, 7504.

[28] T. Canal , N. A. Peppas , J. Biomed. Mater. Res. 1989, 23, 1183. 2808463 10.1002/jbm.820231007

[29] A. Muralidharan , A. C. Uzcategui , R. R. McLeod , S. J. Bryant , Adv. Mater. Technol. 2019, 4, 1900592. 33043126 10.1002/admt.201900592 PMC7546532

[30] C. I. I. Fiedler , E. A. A. Aisenbrey , J. A. A. Wahlquist , C. M. M. Heveran , V. L. L. Ferguson , S. J. J. Bryant , R. R. R. McLeod , Soft Matter 2016, 12, 9095. 27774538 10.1039/c6sm01768a PMC5341082

[31] H. Gojzewski , Z. Guo , W. Grzelachowska , M. G. Ridwan , M. A. Hempenius , D. W. Grijpma , G. J. Vancso , ACS Appl. Mater. Interfaces 2020, 12, 8908. 31961120 10.1021/acsami.9b22272 PMC7033657

[32] V. Birman , L. W. Byrd , in Modeling and Analysis of Functionally Graded Materials and Structures, Vol. 60, American Society of Mechanical Engineers Digital Collection, 2007, pp. 195–216.

[33] L. M. Cox , A. K. Blevins , J. A. Drisko , Y. Qi , Y. Ding , C. I. Fiedler-Higgins , R. Long , C. N. Bowman , J. P. Killgore , Adv. Eng. Mater. 2019, 21, 1900578. 10.1002/adem.201900578 PMC13273708 42317788

[34] L. Diaz-Gomez , B. T. Smith , P. D. Kontoyiannis , S. M. Bittner , A. J. Melchiorri , A. G. Mikos , Tissue Eng. C Methods 2019, 25, 12. 10.1089/ten.tec.2018.0307 PMC6352516 30421648

[35] W. Li , M. Bakhtiary Noodeh , N. Delpouve , J. M. Saiter , L. Tan , M. Negahban , Express Polym. Lett. 2016, 10, 1003.

[36] L. G. Bracaglia , B. T. Smith , E. Watson , N. Arumugasaamy , A. G. Mikos , J. P. Fisher , Acta Mater. 2017, 56, 3. 10.1016/j.actbio.2017.03.030 PMC5544968 28342878

[37] Z. Ling , AMP J. Technol. 1996, 5, 37.

[38] J. C. Kohn , D. M. Ebenstein , J. Mech. Behav. Biomed. Mater. 2013, 20, 316. 23517775 10.1016/j.jmbbm.2013.02.002

