# Supplementary material for: Microscale Photopatterning of Through‐Thickness Modulus in a Monolithic and Functionally Graded 3D‐Printed Part
Source: Small Sci. 2021 Feb 11;1(3):2000017. doi: 10.1002/smsc.202000017 (PMC8388578; doi:10.1002/smsc.202000017)
Supplement: Supplementary file 1 — Supplementary Material [file SMSC-1-2000017-s001.docx]

**Supporting Information**

**
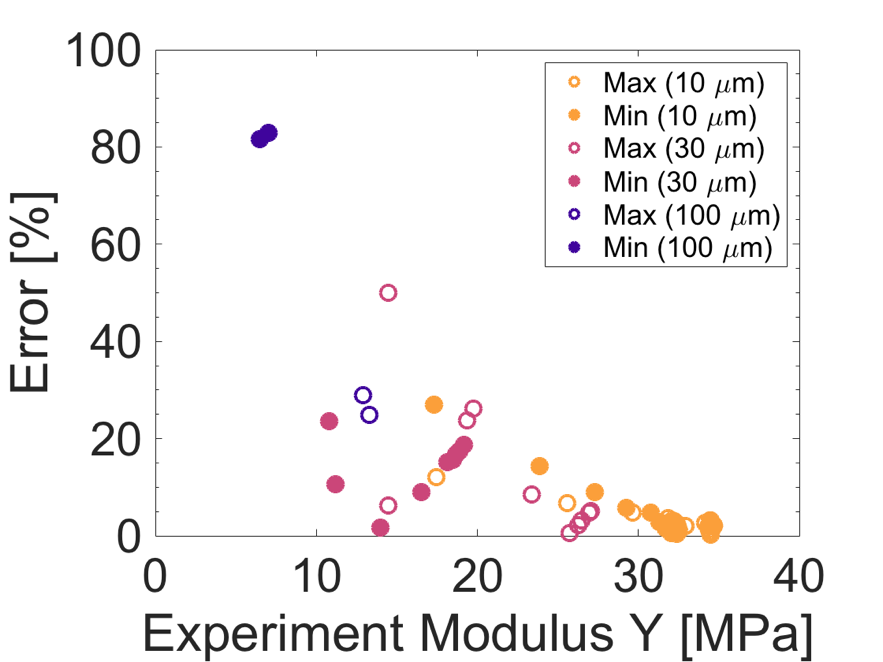
**

Figure S1. Error expressed as a percentage of the absolute difference between modeled and experimental maximum and minimum values for each printed layer divided by the experimental values. Excellent quantitative agreement occurs in high-conversion, high-modulus regions above 20 MPa, but poor agreement occurs in lower-conversion regions below 20 MPa due to localized diffusion effects not accounted for by these bulk predictive curves. The effective exposure was kept constant for all samples *(E** = 82 (mWcm^-2^)^0.77^s*).*


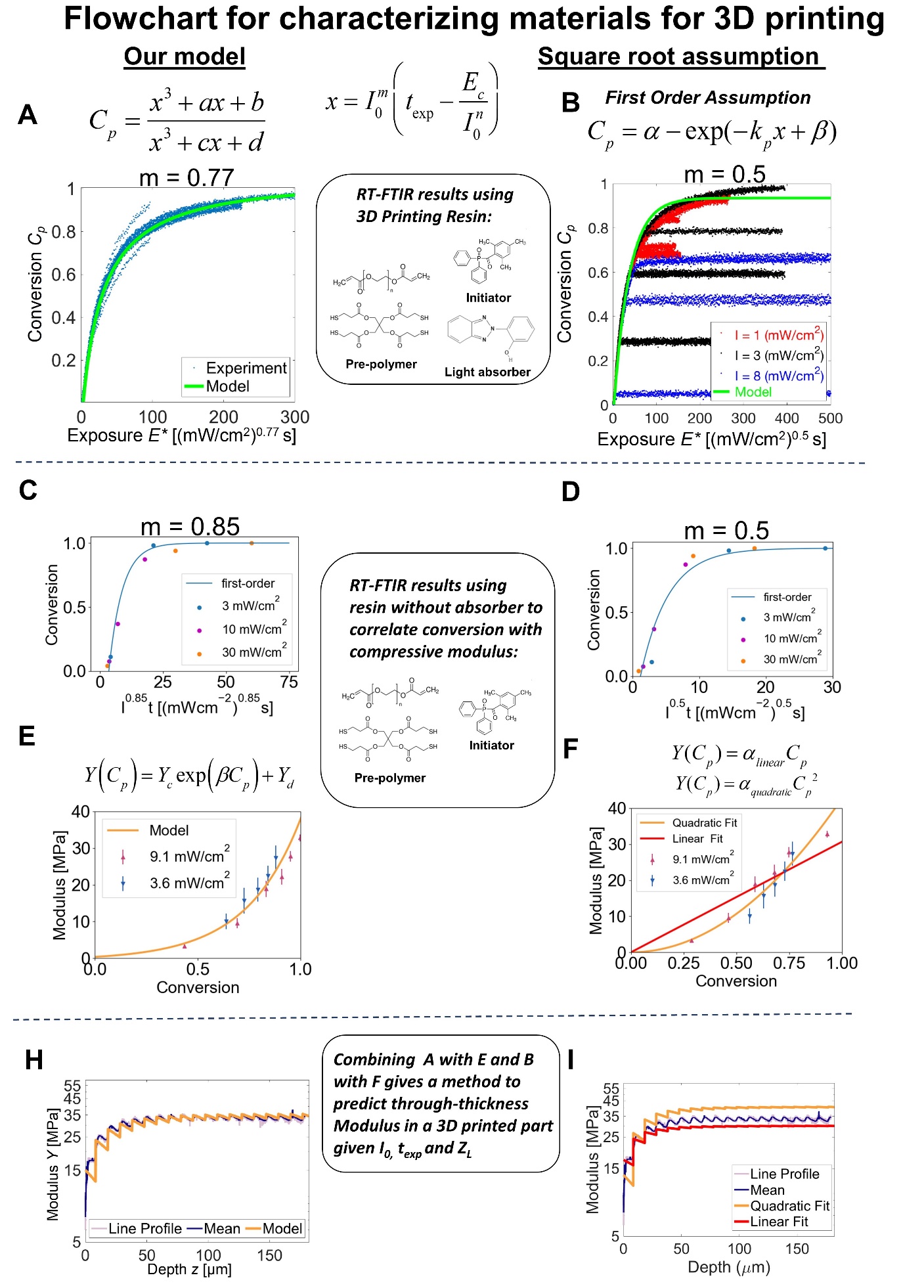


Figure S2. Flowchart to understand methods for describing polymerization kinetics during 3D printing to predict and control properties in depth. A, B) Shown is the double-bond conversion as measured by RT-FTIR for the 3D printing resin. The x-axis is a scaled exposure dose *I_0_^m^t,* where the scaling factor *m* = 0.77 for A and m=0.5 for B. Monomer conversion as a function of exposure conditions can be expressed by a single predictive master curve shown in green, where A shows our empirical model as the master curve and B shows a first order fit derived from the steady-state approximation. C, D) Shown is the double-bond conversion as measured by RT-FTIR for the 3D printing resin without absorber. For both C and D, the master curve is fit to a first-order approximation. However, they differ in that C uses the scaling factor *m* = 0.85 while D uses m = 0.5. E, F) Compressive Young’s modulus of bulk, optically thin layers is plotted against conversion for multiple exposure conditions, yielding a predictive curve (orange). Conversion axis in E and F is found by the master curve in C and D, respectively, causing the data points to differ between models. The modulus data is fit to a linear (red) and a quadratic model (orange) to serve as comparisons to our empirical model. H) Modulus map of a structure 3D printed with *Z_L_* =10 µm overlayed with our model. I) Modulus map of a structure 3D printed with *Z_L_* =10 µm overlayed with a model that assumes a square root dependence on intensity and applies both a linear and quadratic fit to the modulus data.

The model fits were compared to previously established kinetic models that use a square root intensity scaling of m = n = 0.5, and thus do not account for oxygen inhibition (comparison in Figure S2). We find that our model quantitatively agrees in high-conversion and high modulus regions showing less than 15 % error for moduli above 20 MPa and less than 10 % error for moduli above 25 MPa. Low-conversion and low-modulus regions (below 20 MPa) show qualitative agreement (Figure S1). Figure S2 illustrates methods to characterize and predict material properties for 3D printing applications. Because of the need to create optically thin layers for bulk compression testing, we performed RT-FTIR experiments using the 3D printing resin with and without light absorber. Notably, when the light absorber is removed, the resin undergoes significant dark polymerization, therefore affecting the polymerization kinetics described by the master curve. This causes the master curve and scaling factors used in Figure S2A to differ from the master curve and scaling factors used in Figure S2C. The polymerization scaling factors used for our model were found using aforementioned methods for getting the predictive master curve. The scaling factors for the square root assumption (*m* = *n* = 0.5) and the master curve fit remained constant. (Figure S2B and S2D) Figure S2E and S2F show compressive Young’s modulus of bulk, optically thin layers plotted against conversion and compare our model to linear and quadratic fits for the data. The data in Figure S2G and S2H apply the relationships in the previous rows to the multi-layer case which is described by

$$\begin{matrix} I_{layer}(z)=\left\{\begin{matrix} I_{0}\exp[-z\mu] & if & z\geq0 \\ 0 & if & z<0 \end{matrix}\right. \\ I_{total}=\sum_{i=1}^{N} I_{layer}(z-(N-i)T_{z}) \end{matrix}$$

where *I*_0_ is the incident exposure intensity, *µ* is the molar absorptivity constant of the resin, and *N* is the total number of layers in the print. This intensity profile is used to find the conversion *C_p_* throughout the depth of printed parts as,

$$C_{p}(z,t_{\exp})=\frac{x^{3}+ax+b}{x^{3}+cx+d}$$

where

$$x=I_{total}^{m}\left(t_{\exp}-\frac{E_{c}^{*}}{I_{total}^{n}}\right)$$

To investigate the robustness of our approach, we compared our model for modulus and conversion to previously established kinetics models that use a square root intensity scaling (*m* = *n* = 0.5). In every layer thickness case, the mean error of our model is lower than that of a square root assumption. These results highlight the performance of our model in high-conversion and high-modulus regions where the mean absolute error is 1.0 MPa as compared to 5.9 MPa for the square-root modulus model and 3.2 MPa for the linear modulus model when *Z_L_*= 10 μm. (Figure S2I) Overall, our model outperforms other variants used in the field that assume a linear^[1]^ (*n=m=*1) or a square root^[2]^ (*n=m=*0.5) scaling of intensity to predict conversion.


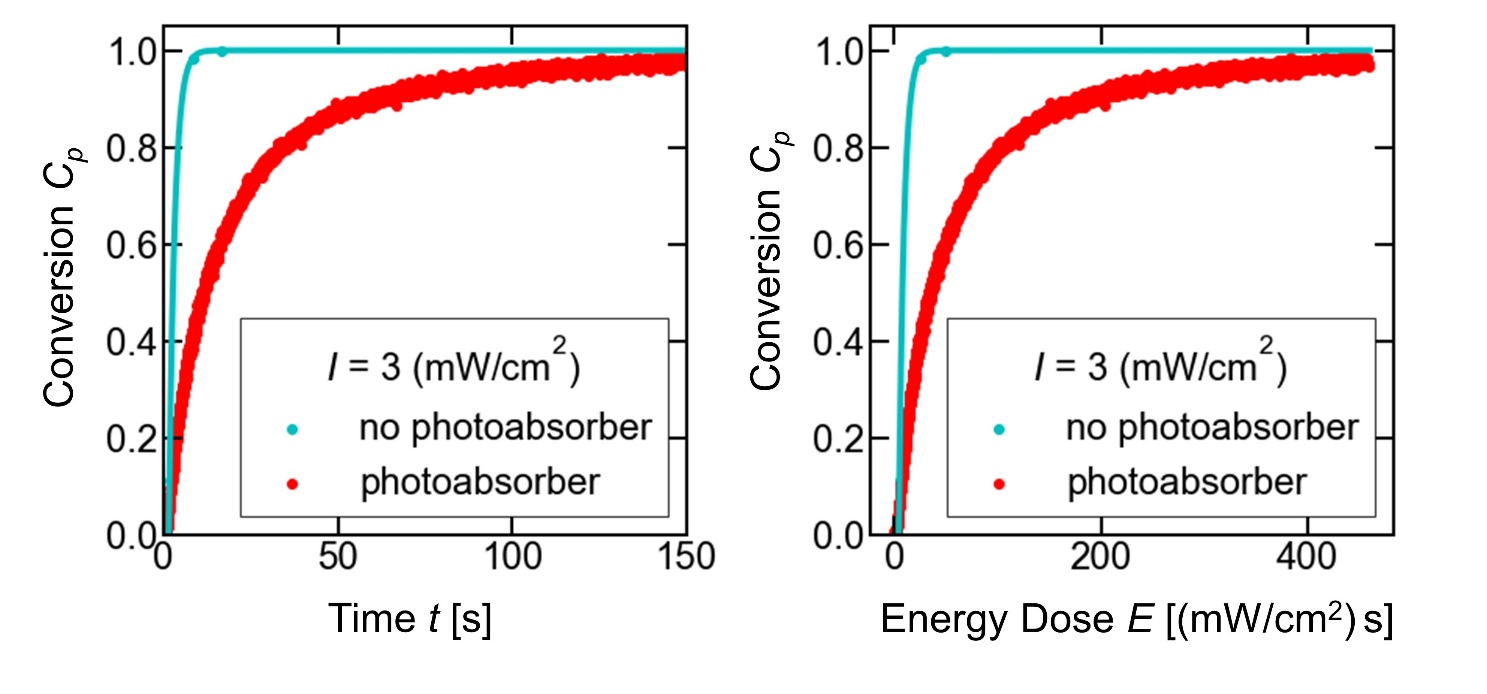


Figure S3. Acrylate monomer conversion as a function of intensity and exposure time as measured by real-time Fourier Transform Infrared (RT-FTIR). Conversion as function of time and energy dose for a single intensity is shown to compare the polymerization kinetics of the resin with the presence of absorber (red) and without the presence of absorber (cyan). To account for the presence of light absorber, we used the mean of the Beer-Lambert exponential decay function to get *I* = 3 mW/cm^2^.

Upon examination of our material’s polymerization kinetics, we found that it is not sufficient to analyze the resin without the presence of photo-absorber. Without absorber, the resin undergoes significant dark polymerization. Dark polymerization was accounted for by measuring the conversion after a defined exposure time and the subsequent dark polymerization. On the contrary, when the resin is evaluated with absorber, there is no dark polymerization and the reaction proceeds at a slower rate highlighted by the slope differences in Figure S3. According to the BASF website, their absorbers may act as radical scavengers, which would explain the difference in kinetics. Therefore, resin without absorber can’t always be used as a proxy to study real-time polymerization kinetics. The intensity value for the resin containing photo absorber was calculated as the mean of the Beer-Lambert exponential decay function.


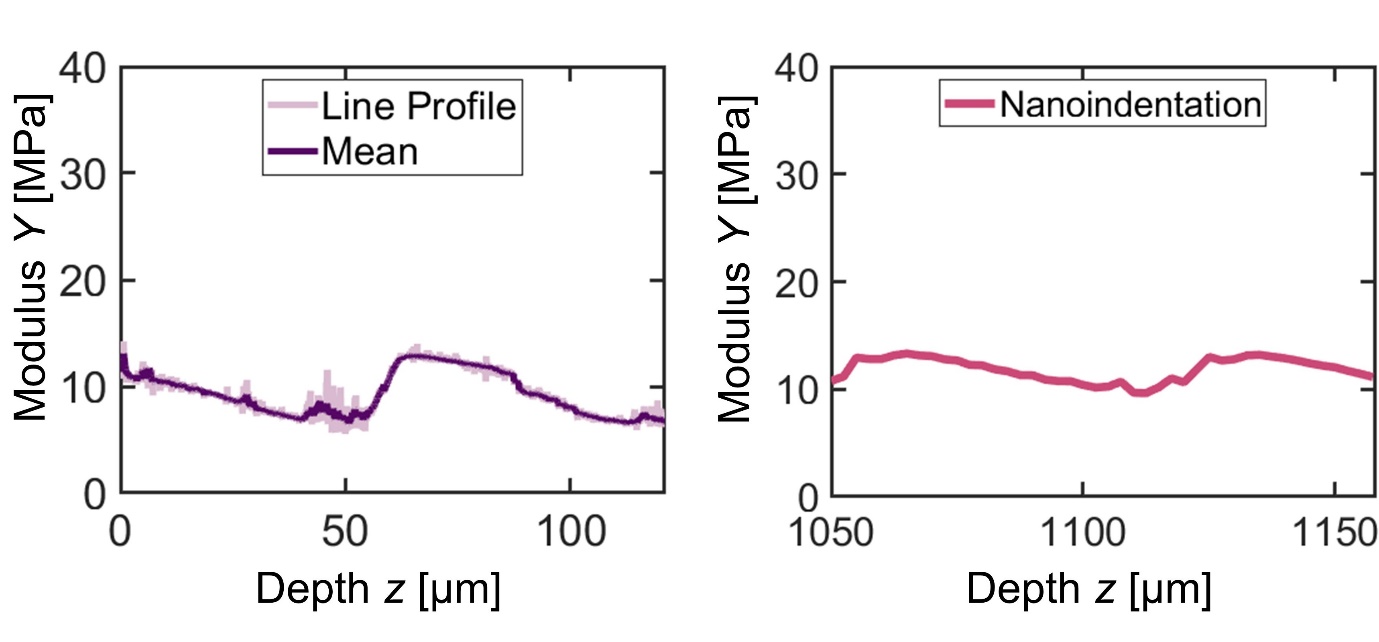


Figure S4. Nanoindentation (NI) results (Right panel), which were taken closer to the middle of the sample, are in agreement with the AFM data (Left panel).

To further investigate the discrepancy between model and experimental modulus at low conversion, we probed the effect of transport and overlapping exposures in a bulk part. Cylindrical structures at *Z_L_* = 10 μm layers with *I*_0_ = 30 mW cm^-2^ and *t*_exp_ = 0.6 seconds were printed and characterized via bulk compression testing. Compressive moduli of the layered samples (26.0 MPa ± 2.4 MPa) closely matched the modulus determined by the AFM (26.3 MPa± 2.9 MPa) for the region with the same exposure conditions.

[1] P. F. Jacobs, D. T. Reid, *Rapid prototyping and manufacturing : fundamentals of stereolithography*; Society of Manufacturing Engineers in cooperation with the Computer and Automated Systems Association of SME, 1992.

[2] J. W. Wydra, N. B. Cramer, J. W. Stansbury, C. N. Bowman, *Dent. Mater.* **2014**, *30*, 605.
